# A Suggestion within Their Means

**Published:** 1919-09-13

**Authors:** 


### WAR MEMORIALS FOR SMALL HOSPITALS.

### A Suggestion Within Their Means.

In a valuable article on the War Memorial Exhibition at South Kensington, a writer in the *Burlington Magazine* for September declares the moral of the exhibits to be that "the humbler the aims of the craftsman the better he succeeds." This should encourage the committees of the smaller hospitals who wish worthily to commemorate the members of the staff who gave their lives in the War. It should also encourage the managers of larger hospitals who, wishing their main memorial to be some addition to the building, yet desire to give due, but not exaggerated, heed to purely artistic considerations. For when the desire of beauty enters, the degree of beauty achieved, no matter on how simple a scale, is the sole criterion of success. Well, when we review the art and craft movement, as the Exhibition, with its division into past and present examples, enables us to do, for all the inspiration of Ruskin and the example of Morris, the fact remains that the greatest success, the real extension of tradition, has been in the humbler crafts—namely, in the lettering of stone, the writing of manuscripts, and the binding of books. We may, therefore, infer that the smaller hospitals, with the least money to spend, have an opportunity to secure beauty in their War memorials, which is peculiarly their own, because the simpler crafts are the most skilfully practised. No small hospital will feel that it has failed in dignity by setting up a stone on which the names of the fallen members of its staff are inscribed in beautiful letters. Nor, in the case of hospitals of moderate size, would it be unworthy that, where the list is too long for engraving on a stone, it should be written in beautiful script on paper or vellum, and the whole, if it covers several leaves, be beautifully bound into a small book. For, after all, the object of these memorials is to commemorate the *names* of the fallen, and the most dignified and simple method of commemoration is to have the names themselves written in beautiful letters. By this means, indeed, the very names are honoured. The cost of such lettering, of such script, is a trifle compared to the beauty of the memorial; and it is only because the lettering with which we are most familiar, that on tombstones, has been left to the monumental mason of commerce, that inscribed slabs of stone have ever seemed blank or dull. But the art of lettering has much good work to its credit where it has been allowed free play outside the field over which the monumental mason is at present supreme—namely, in the field of commemorative inscriptions. There is, therefore, no reason why a hospital, which, with only a few pounds to spend, desires to set such a stone upon its walls, or to have a well-written roll of honour, should be at a loss to know to whose chisel or pen to entrust it. For good "writers" are comparatively numerous, and their names are known. It would be worth while for representatives of the smaller hospitals, although they live in the provinces, to endeavour to visit the Exhibition at South Kensington, of which we would advise them to secure a catalogue. Luckily, however, the stone-lettering of Mr. Eric Gill is famous, and beautiful penmanship has been done by such scribes as Mr. Francis Crease and others. The same is true of bookbinding, of which Miss Katherine Adams' work is well known not only in this country.

Here, then, is a simple, sufficient, and inexpensive way for small hospitals to have a War memorial worthy of the fallen, and, were it adopted, we believe that it would have at least one valuable secondary result. When fine examples of lettering in stone or ink had been once acquired by hospitals, it is probable that a tradition would be created to entrust the writing of future rolls of honour, addresses of presentation, and farewell testimonials to craftsmen of repute. Only by their help can these documents become intrinsically beautiful, and so possess the personal *cachet* which their donors desire.

